# Maximal oxygen uptake, pulmonary function and walking economy are not impaired in patients diagnosed with long COVID

**DOI:** 10.1007/s00421-024-05652-7

**Published:** 2024-11-29

**Authors:** O. K. Berg, N. Aagård, J. Helgerud, M. F. Brobakken, J. Hoff, E. Wang

**Affiliations:** 1 Faculty of Health Sciences and Social Care, Molde University College, Britvegen 2, 6410 Molde, Norway; 2 Treningsklinikken, Medical Rehabilitation Clinic, Trondheim, Norway; 3 Department of Circulation and Medical Imaging, Faculty of Medicine and Health Sciences, Norwegian University of Science and Technology, Trondheim, Norway; 4 Department of Psychosis and Rehabilitation, Psychiatry Clinic, Olavs University Hospital, Trondheim, St Norway

**Keywords:** SARS-CoV-2, Covid-19, Endurance, FEV_1_, Exercise, Late symptoms

## Abstract

**Introduction:**

SARS-CoV-2 may result in the development of new symptoms*,* known as long COVID, a few months after the original infection.

**Purpose:**

It is elusive to what extent physical capacity in patients diagnosed with long COVID is impacted.

**Methods:**

We compared maximal oxygen uptake (V̇O_2max_), one of the single most important factors for cardiovascular health and mortality, expired lung volumes and air flow, oxygen cost of walking and 6-min-walking-test (6MWT), in 20 patients diagnosed with long COVID (11 males and 9 females; 44 ± 16 years (SD); 26.7 ± 3.8BMI, duration of acute phase 1.7 ± 1.2 weeks, tested 4 ± 3 months after long COVID diagnosis) with 20 healthy age and sex matched controls (11 males and 9 females; 44 ± 16 years; 25.9 ± 4.0BMI).

**Results:**

Long COVID patients had a V̇O_2max_ of 41.4 ± 16.2 mL∙kg^−1^∙min^−1^(men) and 38.2 ± 7.5 (women) and this was not different from controls. Similarly, mean spirometry measures in the patient group (VC; FVC; FEV_1_; FEV_1_/FVC) were also not different (85–106%) from predicted healthy values. Finally, inclined treadmill (5%, 4 km∙h^−1^) walking economy was not different between the groups (long COVID: 15.2 ± 1.1 mL∙kg^−1^∙min^−1^; controls: 15.2 ± 1.2 mL∙kg^−1^∙min^−1^), while the 6MWT revealed a difference (long COVID: 606 ± 118 m; controls: 685 ± 85 m; p = 0.036).

**Conclusion:**

V̇O_2max_, oxygen cost of walking, and spirometry measurements did not appear to be impaired in patients diagnosed with long COVID with a prior mild to moderate SARS-CoV-2 infection. The typical outcomes in these essential factors for health and longevity implies that while long COVID can present with a range of symptoms, caution should be made when attributing these symptoms directly to compromised pulmonary function or V̇O_2max_.

## Introduction

Long-term sequelae following a SARS-CoV-2 infection, or COVID-19 disease, is referred to as long COVID. Long COVID is a multimodal condition with patients experiencing different symptoms related to multiple organ systems persisting 3 months after acute infection (Crook et al. 2021; Davis et al. 2023; Ballering et al. 2022). From October 2021, the ICD-10 code U09.9 Post COVID-19 condition, unspecified, has been available to indicate such persisting symptoms (Pfaff et al. 2023). The main symptoms include dyspnea, muscle pain, and exertional intolerance, with substantial impact on quality of life (Njøten et al. 2023; Crook et al. 2021), but an extensive list of up to 200 symptoms may be used for diagnosing (Davis et al. 2023; Wander et al. 2023). Although, the prevalence appears to be higher in patients that have been hospitalized during the acute phase, most infected patients have not been hospitalized, and thus the vast majority with sequelae following COVID-19 are previously healthy individuals that had a mild to moderate course of illness (Davis et al. 2023; Woodrow et al. 2023).

V̇O_2max_ integrates the whole oxygen transport pathway from atmosphere to mitochondria and is widely recognized as one of the single most important factors for endurance exercise capacity and performance, cardiovascular health and mortality. Ribeiro Baptista et al. (2022) found that up to 35% of COVID-19 patients demonstrated an impaired V̇O_2max_ 3 months after hospital discharge. Although some indicate more pronounced reductions in patients with more severe acute phases of the disease (Skjørten et al. 2021; Vonbank et al. 2021), others have documented equal impairments of V̇O_2max_ for mild-moderate, severe and critical COVID-19 patients (Rinaldo et al. 2021). A comprehensive systemic review and meta-analysis by Durstenfeld et al. (2022) found that on average long COVID patients had a 4.9 mL min^−1^ kg^−1^ impairment of their V̇O_2max_. Albeit, not all studies support such a reduction (Fernandes et al. 2023; Wood et al. 2022). Some studies indicate that accompanying the reduction in V̇O_2max_ is a reduction in pulmonary function, such as forced vital capacity (FVC) or expiratory capacity of one second (FEV1), indicating pulmonary limitations (Chamley et al. 2024; Clavario et al. 2021; Ribeiro Baptista et al. 2022). Additionally, impaired mechanical efficiency has been suggested to reduce exercise performance following COVID-19 disease (Pleguezuelos et al. 2021), possibly linked to altered muscle fiber type composition and mitochondrial function of locomotor muscles (Appelman et al. 2024; Jamieson et al. 2024). Notably, the certainty of the results from the meta-analysis by Durstenfeld et al. (2022) were characterized as low, with the authors calling for further investigations into impairment of oxygen uptake and utilization in long COVID patients.

Moreover, of importance, few studies have utilized control groups with individuals not influenced by the COVID-19 pandemic to match with the patients investigated. Interestingly, the limited number of investigations that have made an attempt to control for symptoms in non-infected individuals indicate that the true prevalence of long COVID may be substantially lower than what is observed in uncontrolled studies (Ballering et al. 2022; Hastie et al. 2023). These investigations normalized their findings to time controls that did not have a positive test, and some of the symptoms in the control subjects may still be attributable to societal factors, such as lockdowns, during the pandemic.

The use of outdoor physical activities has a long history in Norway. Importantly, outdoor recreational activities were allowed and strongly encouraged by the health authorities during lockdown periods in Norway. The amount of such activity appears to even have increased during lockdown (Litleskare and Calogiuri 2023). As meeting the activity guidelines by WHO is linked to lower symptom burden for patients with sequelae following COVID-19 (Jimeno-Almazán et al. 2022), outdoor physical activities during lockdown may have blunted the long term impact of SARS-CoV-2 infection in the Norwegian population compared to those living in large urbanized areas (Nigg et al. 2021; Yamada et al. 2023).

The aim of the current study was to investigate exercise capacity and walking performance in patients recently diagnosed with long COVID. These patients were compared to a sex and age matched healthy control group from our clinic where data were collected prior to the COVID-19 pandemic. Additionally, we compared our results to normative values from the Norwegian population. Specifically, we hypothesized that patients with long COVID exhibited 1) reduced V̇O_2max_, walking economy, walking performance, and 2) had impaired pulmonary function, compared to healthy controls and normative data.

## Methods

### Subjects

20 persons (9 females and 11 males aged 24–87 years) volunteered to participate in the study. All participants were recruited from a rehabilitation program at a physical training clinic (Treningsklinikken, Trondheim, Norway) with long COVID as their rehabilitation diagnosis. Nineteen patients received their diagnosis from their general practitioner and one received the diagnosis from a hospital physician. SARS-CoV-2 infection and long COVID characteristics are reported in Table 1. Ten patients had a body mass index (BMI) indicating normal weight while seven were categorized as overweight and three as obese, respectively. In the control group, 19 of the subjects were non-smokers and one was smoker. Average age, height, weight and BMI for both the patient and control groups are presented in Table 2. All participants received written information, and gave their written consent, ahead of the intervention. Before starting any testing or training an examination was performed by a medical doctor with specialization within physical medicine. There was no exclusion based on comorbidity. After inclusion, each patient was age and sex matched to a unique healthy control subject for whom V̇O_2max_, walking economy, and walking performance had been measured in our clinic prior to the COVID-19 pandemic. The person matching patients and controls only had access to participant ID, age, sex, height, and weight, and was blinded to any outcome measures. The age and sex matched control group was healthy with absence of any lung disease. Patients’ physical activity level was recorded using the International Physical Activity Questionnaire—short form (IPAQ-SF). The patients were asked to fill in one form representative for the week prior to their acute phase of COVID-19 and one form for the week prior to reporting for testing in the current investigation. The study was carried out according to the Helsinki Declaration and approved by the Norwegian Regional Committees for Medical and Health Research Ethics.

**Table 1:** Characteristics of patients diagnosed with long COVID

**SARS-CoV-2 infection**	n = 20
**Positive test at time of infection**	20
Hospitalized*	1
* Received outpatient care#*	1
Disease severity of acute phase	
Mild	17
Moderate	2
Severe	1
Symptoms when referred to treatment of long COVID	
Dyspnea	8
Fatigue	19
Heart palpitations/ chest pain	9
Brain fog	7
Dizziness	7
Headache	3
Nausea	1
Myalgia	3
Cough	1
BMI** category**	
Normal weight	7
Overweight	10
Obese	3
Smoking history	
Non-smoker	18
Previous	1
Smoker	1
Comorbidities	
Hypertension	2
Diabetes	1
Myocardial infarction	1
High cholesterol	1
Allergies	4
Fibromyalgia	1
Myalgia	1
Sciatica	1
Autoimmune disease	4
Migraine	3
Chronic headache	1
Tinnitus	2
Benign paroxysmal positional vertigo	1
Current/ previous mental illness	5
Sleeping disorder	1
Hyperkalemia	1
Gastritis	2
Irritable bowel syndrome	1
Glaucoma	1
Number of comorbidities	
0	5
1	3
2	8
3	3
6	1

^*^Hospitalized patient received respirator and non-invasive ventilation treatment

#Visited emergency room but was not admitted to hospital

**Table 2:** Subject characteristics

	All		Males		Females	
	PG(*n* = 20)	CG(*n* = 20)	PG(*n* = 11)	CG(*n* = 11)	PG(*n* = 9)	CG(*n* = 9)
Age (years)	44 ± 16	44 ± 16	51 ± 18	51 ± 18	36 ± 8	37 ± 8
Height (cm)	173 ± 9	177 ± 9	178 ± 6	183 ± 6	168 ± 10	169 ± 6
Weight (kg)	80.6 ± 15.1	81.4 ± 17.9	86.0 ± 10.3	90.9 ± 14.0	75.2 ± 18.7	71.0 ± 17.2
BMI (kg·m^−2^)	26.7 ± 3.8	25.9 ± 4.0	26.9 ± 3.8	26.9 ± 3.2	26.4 ± 4.1	24.5 ± 4.7

Data are presented as mean ± SD. *PG* patient group, *CG* control group

### Pulmonary function

Pulmonary function was measured through spirometry using a Flowscreen spirometer (Jaeger, Germany). The test was performed with three attempts for all participants. The best of three attempts was recorded for FEV_1_, FVC and FEV_1_/FVC. Abnormal spirometry test was defined as less than 80% or greater than 120% of the predicted value.

### Maximal oxygen uptake, walking economy, and walking performance

After performing the spirometry test, the measurements of walking economy were assessed as pulmonary measurements of oxygen uptake when walking on a treadmill (Gymsport 2000, Finland) at 4 km h^−1^ and 5% incline for 5 min. Oxygen uptake was measured through pulmonary measurements, using a Metamax Cortex II Portable System (Cortex Biophysik GmbH, Leipzig, Germany). After the walking economy test, participants progressed directly into a V̇O_2max_ test. The workload progressively increased with ~ 1 km h^−1^ per minute until exhaustion. The recorded respiratory exchange ratio ≥ 1.05 in combination with a plateau in V̇O_2_ were used as criteria for reaching V̇O_2max_ (Wang et al. 2012).

Both walking economy and V̇O_2max_ were calculated as the average of the three highest consecutive 10-s measurements, the last minute of each test. The V̇O_2max_ of the patients was also expressed as a percentage of age and sex reference values for the Norwegian population, previously reported by Edvardsen et al. (2013). The abnormal V̇O_2max_ results were considered for values < 85% of predicted, in accordance with previous investigations in similar patient population (Njøten et al. 2023). Heart rate was recorded during the whole testing procedure using a heart rate monitor (Polar OH1, Finland), where maximal heart rate (HR_max_) was recorded as 3–5 beats ∙ min^−1^ added to the highest observed heart rate during the test.

The 6-min walking test (6MWT) was performed on a separate day and conducted as previously described (AmericanThoracicSociety 2002). The results from the 6MWT where compared to the healthy control group, as well as expressed as a percentage of the predicted walking distance calculated based on age, height, weight, HR_max_ and sex as previously described (Casanova et al. 2011). Less than 85% of predicted values was considered impaired, in accordance with a previous investigation of long COVID patients and 6MWT performance (Huynh et al. 2023).

### Statistical analyses

Statistical analyses were performed using SPSS statistics software 29.0 (IBM, Chicago, IL). The person conducting analysis was blinded for group allocation. Figures were made using GraphPad Prism (GraphPad Software version 7, San Diego, CA). Between group differences were detected using one way ANOVA. Between subject changes in IPAQ-SF was detected using paired samples t-test. For all tests, the level of significance was accepted at *p* < 0.05. Data are presented as mean ± standard deviation (SD).

## Results

### SARS-CoV-2 infection and long COVID diagnosis

The patients reported an average duration of the acute phase of the disease of 1.7 ± 1.2 weeks, time from acute phase to diagnosis of long COVID was 5 ± 6 months and time from long COVID diagnosis to testing for the current study was 4 ± 3 months.

### Maximal oxygen uptake and pulmonary function

**Fig. 1:**
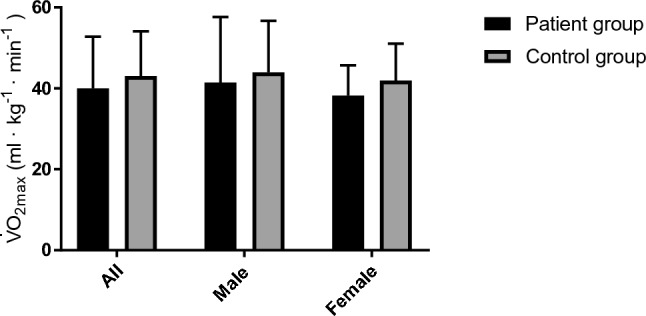
Maximal oxygen uptake (V̇O_2max_) presented as mean ± SD for patients diagnosed with long COVID and a healthy age and sex matched control group

There was no difference between the patient group (PG) and healthy control group (CG) in V̇O_2max_ (PG: 40.0 ± 12.8 vs. CG: 43.0 ± 11 mL∙kg^−1^∙min^−1^), or in the secondary analysis comparing the male (PG: 41.4 ± 16.2 vs. CG: 43.9 ± 12.8 mL∙kg^−1^∙min^−1^) and female (PG: 38.2 ± 7.5 vs. CG: 41.9 ± 9.1 mL∙kg^−1^∙min^−1^) groups (Fig. 1**).** Compared to age and sex reference values (Edvardsen et al. 2013), the patient group displayed 103 ± 27% of predicted values for V̇O_2max_. Two male subjects had a V̇O_2max_ < 85% of predicted (58 and 56%), and one female (80%).

The mean of all spirometry variables was within the normative range of predicted values, see Table 3. Individual patients with a result < 80% of predicted was observed for the average results in VC (*n* = 3), FVC (*n* = 2), FEV_1_ (*n* = 2) and for the best of three attempt only in VC and FVC (*n* = 2 and *n* = 1, respectively). There was no difference in HR_max_ between patients and controls with values of 176 ± 24 vs. 174 ± 44 bpm, respectively. Due to technical issues with the heart rate monitor, HR_max_ was not recorded in two subjects in the control group.

**Table 3:** Spirometry measures in patients diagnosed with long COVID

	All (*n* = 20)		Males (*n* = 11)		Females (*n* = 9)	
	Averag**e**	Best attempt	Average	Best attempt	Average	Best attempt
VC (%)	91 ± 12	93 ± 12	89 ± 12	94 ± 13	85 ± 13	91 ± 10
FVC (%)	100 ± 13	103 ± 12	100 ± 15	104 ± 14	101 ± 10	103 ± 11
FEV_1_ (%)	98 ± 11	100 ± 11	101 ± 13	103 ± 12	95 ± 9	97 ± 10
FEV_1_/FVC (%)	100 ± 11	103 ± 10	103 ± 11	106 ± 11	97 ± 10	100 ± 9

*VC* Vital capacity, *FVC* Forced vital capacity, *FEV*_*1*_ Forced expiratory volume in 1 s. Three measurements were conducted for each measurement and expressed as percentage of predicted value. Average is the sum of the measurements divided by three. Best attempt is the highest value recorded for a variable from three measurements. Data are presented as mean ± SD and as % of predicted values

### Walking economy and walking performance

**Fig. 2:**
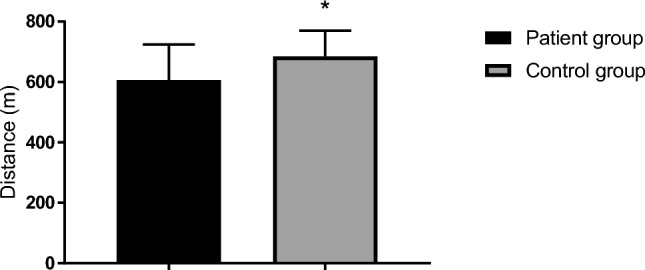
Submaximal endurance performance: Distance covered during a six-minute walk test, presented as mean ± SD for patients diagnosed with long COVID and a healthy age and sex matched control group. * *P* < 0.05

There was no difference between patients and healthy controls in the oxygen cost of walking at standard submaximal workload (PG: 15.2 ± 1.1 vs. CG: 15.2 ± 1.2 mL∙kg^−1^∙min^−1^). However, with regards to walking performance the patients covered less distance (606 ± 118 m) compared to the healthy controls (685 ± 85 m) during the 6MWT (p = 0.036, Fig. 2**)**. The patients’ distance was 115 ± 19% of predicted. Two patients (one due to dizziness and exhaustion, and one due to joint pain following the treadmill test) and four healthy controls (did not show up the day when the test was conducted), did not complete the 6MWT. One patient performed < 85% of predicted (84.8%). This patient was the same that had impaired VC and FVC, and had a 58% of predicted V̇O_2max_.

### Physical activity

From the IPAQ-SF the patient group reported an activity level prior to their acute phase of COVID-19 of 3177 ± 2077 MET minutes/week, and on time of testing 1981 ± 2254 MET minutes/week, which indicated a trend towards reduced overall activity level (*p* = 0.065). The change was mainly driven by a decline in moderate activity from 1182 ± 1521 to 391 ± 492 MET min/ week (p = 0.04), while no significant change was found for vigorous or walking activity (1147 ± 765 to 1056 ± 1887 and 848 ± 750 to 534 ± 842 MET min/ week, respectively).

## Discussion

The aim of the current study was to investigate physical capacity, assessed as V̇O_2max_, pulmonary capacity, and walking economy. The main findings were that (1) V̇O_2max_ in patients diagnosed with long COVID was not reduced compared to healthy controls, (2) pulmonary function, measured as VC, FVC, FEV_1_, and FEV_1_/FVC was within normal range, and (3) walking economy was similar between patients with long COVID and healthy controls, albeit 6-min walking performance was reduced in the former group. Taken together, our results reveal no impairments in aerobic endurance or pulmonary function in patients diagnosed with long COVID, suggesting that oxygen supply and utilization may not be attenuated or directly connected with the diverse symptom burden of long COVID.

### Long COVID and V̇O_2max_

Our result, indicating that V̇O_2max_ was not impaired, is in contrast to most previous studies examining V̇O_2max_ in long COVID patients that have, on average, documented a reduction of 4.9 mL∙kg^−1^∙min^−1^ in this population (Durstenfeld et al. 2022). One of few studies that included a control group, by comparing the V̇O_2max_ between non-hospitalized patients with and without long-term symptoms, reported as much as 8.2 mL∙kg^−1^∙min^−1^ lower V̇O_2max_ in the patients with long COVID 8 ± 2 months after onset of acute COVID-19 illness (Colosio et al. 2023). However, the two groups were not evenly matched. Of note, the mean age in the long COVID patients was 5 years older than the group without persisting symptoms. Moreover, the proportion of females was 64% in the long COVID group and 50% in the control group. As, V̇O_2max_ may decline by ~ 8% per decade and females on average have 33% lower V̇O_2max_ compared to males (Edvardsen et al. 2013), such differences between groups are relevant. Interestingly, another study separating long COVID patients from those fully recovered after SARS-CoV-2 infection found no significant difference in V̇O_2max_ for any of the groups compared to predicted values. Yet, the V̇O_2max_ of the patients was lower than healthy controls, due to their value being 130% of predicted (Beaudry et al. 2022). In the current study, our finding of maintained V̇O_2max_ is strengthened by both the inclusion of a sex and age-matched reference group without previous SARS-CoV-2 infection and a comparison to normative population data (Edvardsen et al. 2013). In contrast to the current investigation, studies solely using reference values for V̇O_2max_ should be interpreted with some caution as reference values obtained by voluntary participation in strenuous exercise tests may be skewed by selection bias towards individuals more inclined to be active and physically fit. Furthermore, reference studies largely report data on healthy participants, excluding people with chronic disease or other impairments. As persisting dyspnea, fatigue and cognitive symptoms 4 months after the acute phase of COVID-19 may occur more frequently in people with pre-existing comorbidities (Iversen et al. 2024), it may not be a fair comparison to evaluate if long COVID has induced a decline in patients V̇O_2max_ using reference values of healthy people without comorbidities motivated to conduct a V̇O_2max_ test.

Some nation-specific factors may potentially explain our findings: (1) Throughout the pandemic it was a strong encouragement from the government to exercise by engaging in outdoors activities, also in the period of lockdown; (2) Norway has a relatively small population of ~ 5 million inhabitants, demographically spread out, which facilitated daily exercise without the risk of violating the regulations of social distancing; (3) Nation-specific easy access, with few restrictions, to mountains, forests, long coastline, trails, even in most towns and cities. In fact, outdoor recreational activity was reported to have increased by 291% during lockdown for citizens in Oslo, the capital of Norway (Venter et al. 2020). This may be important as the main factor linked to impaired V̇O_2max_ has been suggested to be general deconditioning and not related to pulmonary function (Jahn et al. 2022). Although the long COVID patients in the current investigation tended to decrease their total activity level after the acute phase of the illness, the results from IPAQ-SF indicate that they maintained an average weekly activity within typical range for their age compared to pre-pandemic studies in Norway (Dyrstad et al. 2014; Moseng et al. 2014). Thus, it is possible that the long COVID group did not suffer sufficient deconditioning to decrease their V̇O_2max_. In a study that managed to include a non-infected time control group, Fernandes et al. (2023) demonstrated that although V̇O_2max_ appeared to be reduced following SARS-CoV-2 infection in patients with cardiorespiratory disease, the reduction was not different from that in patients whom did not contract COVID-19. Furthermore, endurance trained persons, with long COVID, have even demonstrated values of V̇O_2max_ > 80 mL∙kg^−1^∙min^−1^ (Meloni et al. 2023), indicating that the link between symptoms of long COVID and aerobic capacity may not be that apparent. Thus, if deconditioning could be avoided through maintenance or increase of recreational activity, an un-impaired V̇O_2max_ could be expected, regardless of SARS-CoV-2 infection and development of potential long COVID.

### Long COVID symptoms, comorbidities and V̇O_2max_

V̇O_2max_, which is dependent on all steps in the O_2_ pathway from atmosphere to mitochondria and widely considered the most important factor of aerobic endurance performance (Bassett and Howley, 2000), as well as a key factor for health and longevity (Myers et al. 2002; Ross et al. 2016), was not reduced in patients with long COVID. Both when comparing to our local reference group not influenced by the COVID-19 pandemic and to normative data from the Norwegian population. This is somewhat surprising as many of the symptoms reported by the patients such as fatigue, heart palpitations and dyspnea would intuitively indicate metabolic or cardiovascular limitations. However, when examined in the general Norwegian adult population prior to the COVID-19 pandemic, feeling of fatigue was the most common symptom with a prevalence of approximately 60% reporting ≥ 1 on a scale of 0 to 10 (where 0 is “not present” and 10 is “as bad as you can imagine”) and 35% responding to the question with ≥ 3 (Krogstad et al. 2020). Moreover, in the same study 45% reported 1–2 comorbidities and 13% three or more. In comparison, 55% of the patients in the current investigation reported 1–2 comorbidities and 20% three or more. Thus, the number of comorbidities is somewhat above the general population and given that it has been found to be associated with higher level of symptoms such as fatigue, one could expect that feeling of fatigue also would be high in the patient group. This is apparent with 95% of the patients having fatigue as one of the main symptoms related to their referral for long COVID treatment. Given the prevalence of symptoms in the normal population prior to the pandemic it could be questioned if symptoms such as fatigue, drowsiness and pain, at least for all patients, is directly linked to the SARS-CoV-2 infection. Notably, there is substantial overlap between the symptoms that were reported as cause for referral to treatment of long COVID and those normally associated with the comorbidities present prior to SARS-CoV-2 infection in the patient group (see Table 1).

An extensive list of up to 200 symptoms across multiple organ systems has been linked to long COVID diagnosis (Davis et al. 2023; Crook et al. 2021). This constitutes a substantial challenge when holding a previous SARS-CoV-2 infection responsible for a reduction in V̇O_2max_ as only some symptoms are associated with the cardiovascular system and its ability to maximally transport oxygen from air to mitochondria. Thus, in the current study, the patients with long COVID may suffer from factors that do not influence V̇O_2max_. Interestingly, a long COVID diagnosis shares many symptom-similarities with people diagnosed with myalgic encephalomyelitis (ME) and chronic fatigue syndrome (CFS). Patients with these diagnoses are also generally documented to have a reduced V̇O_2max_ compared to healthy controls of 5.2 mL∙kg^−1^∙min^−1^ (Franklin et al. 2019), that interestingly is remarkably similar to that reported in long COVID patients (Durstenfeld et al. 2022). Thus, it may be questioned if many previous observations of a reduced V̇O_2max_ in long COVID are diagnosis-specific. Indeed, few studies have addressed confounding factors and even fewer have included a control group where it has been adjusted for long COVID symptoms (Barbagelata et al. 2022; Durstenfeld et al. 2023). It may very well be that previously reported reductions in V̇O_2max_ are not a direct result of a SARS-CoV-2 infection.

### Long COVID, V̇O_2max_, and pulmonary capacity

Individuals in the current study exhibited a typical V̇O_2max_ for their age and sex. As V̇O_2max_ reflects integration of all oxygen transport steps and oxidative capacity (Wagner, 2000), it is unsurprising that pulmonary capacity also revealed values in the normative range. SARS-CoV-2 infection has previously been shown to impair pulmonary function (Blanco et al. 2021), even lasting for at least a year (van Willigen et al. 2023). Albeit these findings appear to be more prevalent in severe disease and for patients with several comorbidities (Lam et al. 2022; Chamley et al. 2024). In the present study, all but one of the patients diagnosed with long COVID had a history of mild to moderate infection, less likely to cause lasting damage to lungs, and this may explain why no reduction in pulmonary function was detected. Although, FVC, FEV_1_ and FEV_1_/FCV ratio in a previous investigation of long COVID patients were observed to be within normal range of predicted values, unadjusted regression analysis indicated an association between FEV_1_ and V̇O_2max_ (Njøten et al. 2023), suggesting that lung function may play a role in exercise limitations for this group. Indeed, another study that identified reductions in V̇O_2max_ in long COVID patients simultaneously identified reductions in FVC and FEV_1_ (Chamley et al. 2024). On the other hand, Holley et al. (2024) found no association between FVC or FEV_1_ with V̇O_2max_, despite a substantially impaired V̇O_2max_ in young patients with persisting dyspnea ~ 11 months post SARS-CoV-2 infection. Similarly, Colosio et al. (2023) found no difference for FVC and FEV_1_ between long COVID patients and recovered controls without long term symptoms, despite V̇O_2max_ being substantially reduced in the long COVID group.

Importantly, the patients in the current study did not have any known other lung disease, such as chronic obstructive pulmonary disease, reducing the risk for lung damage. However, it should be noted that two of the subjects had results below 80% of predicted for their best of three attempts in VC (68% and 72%) and one of these also had an FVC of 77%. Yet, none of them had atypical results for FEV_1_ or FEV_1_/FVC. These two subjects also had a V̇O_2max_ of < 60% of predicted values and, of importance, were relatively old (86 and 78 years) and had at least two comorbidities. One of the subjects was also the only patient in this study to be hospitalized during the acute phase of their SARS-CoV-2 infection, suggesting that severe disease is more likely to result in reduced V̇O_2max_ and impaired pulmonary function.

### Long COVID, walking economy, and walking performance

Another important factor for aerobic endurance is walking economy and this factor was also similar between patients with long COVID and healthy controls. As walking economy is defined as the oxygen cost at a given submaximal workload, it strengthens the assumption that aerobic endurance, overall, from submaximal to maximal aerobic metabolism, was not impaired in patients with long COVID in the present study. Our result is in accordance with a previous study that documented similar oxygen cost at submaximal cycle-ergometer work rates of long COVID patients and COVID-19 naive controls (Beaudry et al. 2022). The similar work economy strengthens the assumption that patients with long COVID, in general, had a typical oxygen supply and demand.

The measurements of physiological components of aerobic endurance were, however, not reflected in walking performance in the present study as the 6MWT revealed a ~ 12% reduction in walking distance compared to the healthy control group. As performance is a result of many factors, including non-physiological factors, it is difficult to know what may have caused the reduction, but it is in line with previous literature that has documented similar reductions (Beyer et al. 2023). Albeit, Wood et al. (2022) found no impairment in 6MWT distance in patients with persisting cardiac symptoms one year following acute SARS-CoV-2 infection. Notably, only a moderate correlation has been described between oxygen cost of walking and 6MWT performance, while a strong correlation was reported with self-reported severity of fatigue and 6MWT performance (Barbosa et al. 2016). This indicates that factors other than the oxygen supply and utilization in muscles, such as perceived symptoms, may also account for the observed difference in the walking distance between the long COVID and healthy controls in the current study. Finally, it should be noted that patients with long COVID in the current study, despite walking a shorter distance than the healthy control group, exhibited a 6MWT performance with 115 ± 19% of predicted values, when compared with reference standards from seven other countries, prior to the COVID-19 pandemic (Casanova et al. 2011).

### Study limitations

Although the age and sex matched control group in the current investigation was similar to the long COVID group with respect to obesity and smoking status, we do not have data on number of comorbidities or presence of relevant symptoms in everyday life. Such data would be beneficial to further investigate the etiology of long COVID and its impact on the population. Moreover, as there are no clear objective measures used for diagnosis of long COVID it is possible that studies from different populations vary substantially in inclusion criteria without it being apparent from the reported diagnosis studied. It is also possible that the myriad of symptoms stem from different impairments and that the diagnosis is currently not specific enough as to what part of the physiology is affected. Of note, static lung volumes and lung diffusion capacity were not measured in the current investigation. Some, but not all, studies that have documented impaired V̇O_2max_ in long COVID find reduced diffusion capacity (Chamley et al. 2024; Colosio et al. 2023; Holley et al. 2024; Njøten et al. 2023). Moreover, diffusion capacity appears to improve over time in patients recovering from SARS-CoV-2 infection, except for those that demonstrate exercise ventilatory inefficiency (Dorelli et al. 2024). This indicates that both resting measures of lung diffusion capacity and assessment of ventilatory efficiency during exercise may be relevant measures to include in future investigations. The sample size is also modest in the current investigation and 95% of our subjects had a mild to moderate acute phase and were not hospitalized. As such our data are limited to patients with a mild to moderate SARS-CoV-2 infection, and generalizability to patients with more severe acute phase and intensive care at hospital is low. However, as many as 83% and 90%, respectively, of patients with long COVID do not have a history of hospitalization during their acute phase (Meza-Torres et al. 2022; Aziz et al. 2023), indicating that our findings are relevant to the largest section of this patient group. The studies, including pulmonary assessment of V̇O_2max_ and pulmonary function, with a well-defined control group, in other populations are sought after, along with investigations examining the plasticity of these measures following exercise training.

## Conclusion

In long COVID patients with a previous mild to moderate SARS-CoV-2 infection, V̇O_2max,_ walking economy, and pulmonary function, key factors for health and longevity, were similar with what is typically observed in the healthy population. The weekly activity level also remained within typical range. A broad spectrum of symptoms describes patients with long COVID and caution should be made when attributing long COVID, and its symptoms, directly to compromised pulmonary function or V̇O_2max_.
